# The impact of xanthine oxidase (XO) on hemolytic diseases

**DOI:** 10.1016/j.redox.2018.101072

**Published:** 2018-12-10

**Authors:** Heidi M. Schmidt, Eric E. Kelley, Adam C. Straub

**Affiliations:** a Pittsburgh Heart, Lung, Blood and Vascular Medicine Institute, United States; b Department of Pharmacology and Chemical Biology, University of Pittsburgh, Pittsburgh, PA, United States; c Department of Physiology and Pharmacology, West Virginia University, School of Medicine, Morgantown, WV, United States

**Keywords:** Hemolysis, Heme toxicity, Xanthine oxidase, Reactive oxygen species, Therapeutics

## Abstract

Hemolytic diseases are associated with elevated levels of circulating free heme that can mediate endothelial dysfunction directly via redox reactions with biomolecules or indirectly by upregulating enzymatic sources of reactive species. A key enzymatic source of these reactive species is the purine catabolizing enzyme, xanthine oxidase (XO) as the oxidation of hypoxanthine to xanthine and subsequent oxidation of xanthine to uric acid generates superoxide (O_2_^•-^) and hydrogen peroxide (H_2_O_2_). While XO has been studied for over 120 years, much remains unknown regarding specific mechanistic roles for this enzyme in pathologic processes. This gap in knowledge stems from several interrelated issues including: 1) lethality of global XO deletion and the absence of tissue-specific XO knockout models have coalesced to relegate proof-of-principle experimentation to pharmacology; 2) XO is mobile and thus when upregulated locally can be secreted into the circulation and impact distal vascular beds by high-affinity association to the glycocalyx on the endothelium; and 3) endothelial-bound XO is significantly resistant (> 50%) to inhibition by allopurinol, the principle compound used for XO inhibition in the clinic as well as the laboratory. While it is known that circulating XO is elevated in hemolytic diseases including sickle cell, malaria and sepsis, little is understood regarding its role in these pathologies. As such, the aim of this review is to define our current understanding regarding the effect of hemolysis (free heme) on circulating XO levels as well as the subsequent impact of XO-derived oxidants in hemolytic disease processes.

## 1 Introduction

Excess circulating free heme is associated with numerous hemolytic diseases including sickle cell disease (SCD), thalassemia, sepsis, cardiac bypass, and malaria [1], [2], [3], [4], [5]. Oxidation of reduced heme (Fe^2+^ → Fe^3+^) can result in heme and hemoglobin (Hb) release from red blood cells (RBCs) into the circulation [2], [6]. Under normal physiologic conditions, heme and Hb are immediately scavenged and degraded in plasma or sequestered and transported to tissues such as the liver for degradation [2], [6]. However, under conditions of severe hemolysis, circulating heme reaches levels that saturate the scavenging and degradation pathways, resulting in significant extracellular levels of free heme that can initiate intravascular cell and tissue damage [2], [6].

**Fig. 1:**
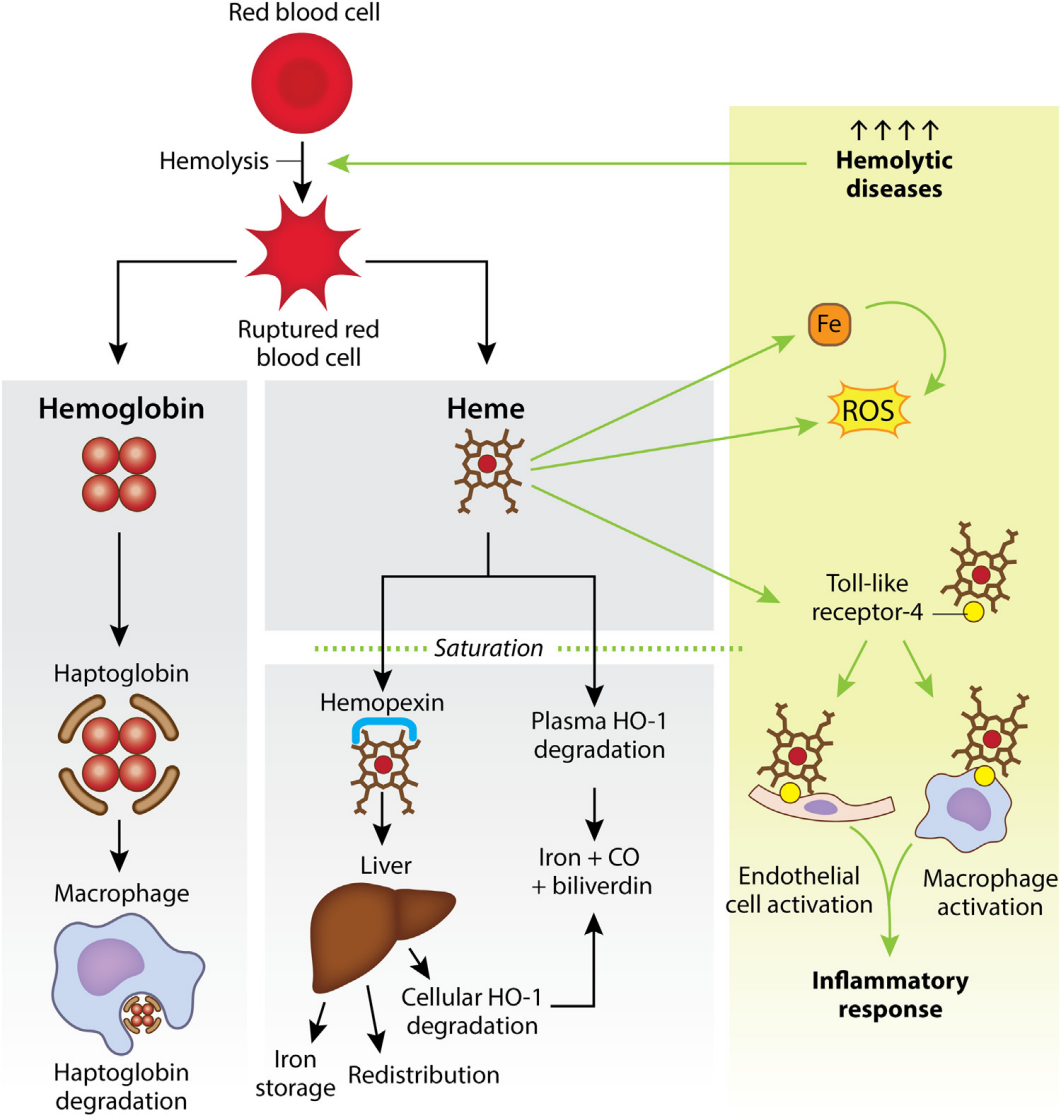
A red blood cell (RBC) undergoing hemolysis releases hemoglobin (Hb) and heme. Hb is bound by haptoglobin (Hp) and targeted to macrophages for degradation. Heme is either 1) bound by hemopexin (Hx) and targeted to the liver for iron storage, redistribution, or degradation by cellular heme oxygenase-1 (HO-1); or 2) degraded directly in the plasma by HO-1 into iron, carbon monoxide (CO) and biliverdin (gray panel). Hemolytic diseases cause elevated levels of hemolysis which saturate Hx and HO-1. When these pathways are saturated, heme activates toll-like receptor-4 (TLR4) triggering an immune response. Separately, heme can also generate reactive oxygen species (ROS) directly and indirectly through iron (green panel).

Under basal levels of hemolysis, Hb is bound by haptoglobin (Hp) and targeted to macrophages where the complex is taken up via endocytosis and degraded (Fig. 1) [2]. Likewise, free heme in the plasma is either bound by hemopexin (Hx) or immediately degraded to iron, carbon monoxide and biliverdin by the enzyme heme oxygenase-1 (HO-1) [1]. Hx targets heme primarily to the liver and spleen, allowing heme to be cleared through the endothelium, endocytosed by hepatocytes and degraded by cellular HO-1 or redistributed for heme iron recycling (Fig. 1) [2], [6]. However, under conditions of severe hemolysis, Hx and HO-1 become saturated, resulting in oxidative damage to surrounding tissues due to a decrease in heme degradation and simultaneous increase in circulating free heme [2]. For example, reaction of free heme with O_2_ can generate superoxide (O_2_^•-^), and subsequently elevate hydrogen peroxide (H_2_O_2_) levels via spontaneous or enzymatic dismutation as well as hydroxyl radical (HO^•^) levels by reaction of peroxide(s) with transition metals including heme-iron (Fe). Enhanced abundance of these reactive species mediate lipid, protein, and DNA oxidation resulting in cell and tissue damage, endothelial dysfunction, and loss of vascular homeostasis (Fig. 1) [1], [7]. While the catalytically-active Fe in heme as well as “free Fe” derived from heme is considered the seminal source of oxidants in hemolytic disease, subsequent contributions from alternative sources are significant to the progression of this inflammatory process. For example, free heme activates toll-like receptor 4 (TLR4) signaling, resulting in activation of pro-inflammatory pathways which include amplification of ROS levels from sources alternative to those generated via Fe-mediated reactions (Fig. 1) [2], [8].

In addition to hemolysis, reactive oxygen species (ROS) and reactive nitrogen species (RNS) (e.g. nitric oxide (^•^NO), nitrogen dioxide (^•^NO_2_) and peroxynitrite (ONOO^-^)) are generated under numerous pathological conditions that often accompany hemolytic diseases (e.g. ischemia-reperfusion injury and chronic inflammation) [9], [10], [11]. The main pathways in which O_2_^•-^ is generated include the mitochondrial electron transport chain, nicotinamide-adenine dinucleotide phosphate (NADPH) oxidases, uncoupled nitric oxide synthase (NOS), and xanthine oxidase (XO) [10]. XO is one such mechanism that is noted to be upregulated in hemolytic disease [12]. XO generates oxidants by shuttling electrons derived from purine oxidation to either univalently (O_2_^•-^) or divalently (H_2_O_2_) reduce O_2_ [12]. This elevation of XO activity may result in increased formation of RNS via the diffusion-limited reaction between XO-derived O_2_^•-^ with ^•^NO to generate ONOO^-^ [13]; however, increased rates of O_2_^•-^ formation from the other sources mentioned above may also contribute. The increased presence of ONOO^-^ may lead to alteration in cell signaling via post-translational modification of critical sulfhydryls on effector proteins, diminution of ^•^NO-mediated vasodilatory action, and loss of endothelial barrier integrity via induction of membrane lipid peroxidation [14], [15].

## 2 Xanthine oxidase

Xanthine oxidoreductase (XOR) is a name commonly used to encompass two interconvertible forms of the same enzyme: dehydrogenase (XDH) and oxidase (XO) [16]. XOR is transcribed and translated as XDH, a ~300 kDa homodimer consisting of four redox centers in each subunit: one molybdenum cofactor (Mo-co), one flavin adenine dinucleotide (FAD) site, and two Fe_2_S_2_ sites [17]. XDH catalyzes the oxidation of hypoxanthine to xanthine and xanthine to uric acid at the Mo-co site and electrons are shuttled via the two Fe_2_S_2_ clusters to the FAD site where NAD^+^ is reduced to NADH. XOR exists primarily as XDH intracellularly; yet, once in the extracellular space and circulation XO is the dominant isoform [17]. The principle difference between XO and XDH is their oxidizing substrate affinity where XO demonstrates diminished affinity for NAD+ and over 11-fold increased affinity for O_2_ [18]. As such, electrons derived from hypoxanthine and xanthine oxidation by XO are quickly accepted by O_2_ to generate O_2_^•-^ and H_2_O_2_ [12]. Della Corte and Stirpe made several important discoveries in the late 1960s and early 1970s showing that XOR is irreversibly or reversibly converted to XO via proteolysis or oxidation of cysteine residues, respectively [19], [20], [21]. As the crystal structure of bovine milk XDH was solved, the site of proteolysis was identified as following Lys551 and the sites of oxidation were identified as Cys535 and Cys592 with formation of a disulfide bridge [18].

## 3 XOR regulation

While much is known regarding XOR biochemistry, transcriptional and translational regulation is much less understood. The human *xdh* gene is located on the p22 band of chromosome two and contains several possible binding sites for translational modification: four CCAAT/enhancer binding sites, three IL-6 responsive elements, an NF-κB site, and TNFα, interferon-γ, and interleukin-1 responsive units [22]. XOR expression is reported to be controlled by a variety of factors including hormones, growth factors, and inflammatory cytokines; yet, the most studied effector of XOR expression/activity is hypoxia [23], [24]. While many studies have described transcriptional and post-translational up-regulation of XOR by severe hypoxia [25], [26], [27], [28], modest hypoxia (10% O_2_) is also capable of inducing significant elevation of XOR expression, activity, export from endothelial cells, and XO-dependent ROS production [24]. The described moderate hypoxic conditions are comparable to levels observed in congestive heart failure patients [24]. Parks and Granger were the first to describe an elevation in purine catabolites under hypoxic conditions as they attributed the increased hypoxanthine observed during hypoxia to the breakdown of ATP→ADP→AMP→adenosine→inosine→hypoxanthine (purine degradation pathway) [29]. This is crucial in terms of XO function as increased levels of hypoxanthine require increased XO activity for further oxidation to uric acid, all while generating O_2_^•-^ and H_2_O_2_ as byproducts [29]. It is important to note that elevated circulating free heme can induce RBC lysis resulting in release of ATP into the circulation [30], [31], [32]. This ATP is quickly catabolized to adenosine then to hypoxanthine creating a milieu similar to that described above for moderate hypoxia [30]. Elevated levels of hypoxanthine may consequently trigger upregulation of XOR activity in addition to activating purine salvage via hypoxanthine-guanine phosphoribosyltransferase (HPGRT) [33]. Interestingly, we have previously described upregulation of XOR via adenosine activation of adenosine A_2B_ receptors on endothelial cells [24]. Therefore, the combination of increased adenosine and hypoxanthine levels could significantly amplify XOR activity and allied ROS generation during hemolytic crisis; a process already wrought with abundant oxidant generation attributable to heme itself.

## 4 XOR-endothelial interaction

A critical concept when considering the impact of XOR on disease processes in general and hemolytic disease specifically, is that XOR is mobile and has a high affinity (*K*_*d*_ = 6 nM) for glycosaminoglycans (GAGs) on the apical surface of the vascular endothelium [34], [35]. As such, XOR can be upregulated in one anatomic site (e.g. liver), exported to the circulation, bound to endothelial GAGs and thus sequestered in vascular beds distal from the site of origin [34], [35], [36], [37], [38]. When coupled to the elevated circulating levels of hypoxanthine, it is in this setting that XO can critically contribute to oxidant-mediated vascular dysfunction [39]. Binding and immobilization of XOR on the vascular GAGs also has significant kinetic consequences including alteration in the relative amounts of O_2_^•-^ and H_2_O_2_ produced as well as resistance to inhibition by pyrazolopyrimidine-based inhibitors (see Therapeutic Inhibitors of XO, below) [40], [41], [42]. In the aggregate, mobility and capacity to avidly associate to the endothelium in a manner that is resistant to inhibition affords XO the ability to critically contribute to loss of vascular homeostasis.

## 5 Therapeutic inhibitors of XO

There are currently two XO inhibitors that are FDA approved for the treatment of gout: allopurinol and febuxostat [43]. While allopurinol has been used in the clinic for over fifty years, febuxostat (Uloric^®^) was approved in 2009 [43]. Current reports suggest allopurinol may be the superior drug for diminishing uric acid levels to the extent that symptoms of gout are alleviated; however, febuxostat is a more potent XO inhibitor and may be more useful in treating diseases with elevated XO activity at the surface of endothelial cells [43]. Allopurinol is a hypoxanthine mimetic that prevents oxidation of hypoxanthine and xanthine at the Mo-co site of XOR [44]. XOR oxidizes allopurinol, a suicide inhibitor, with the transfer of two electrons to form oxypurinol, the pharmacologically active form of the drug, which then competitively inhibits XO [40], [45]. Since the oxidation of allopurinol to oxypurinol results in reduction of XOR, O_2_^•-^ and H_2_O_2_ are generated as unfortunate byproducts [40]. In addition, GAG-immobilized XOR is resistant to inhibition by allo/oxypurinol. For example, concentrations (200–400 µM) of allo/oxypurinol, greater than the clinical working range (20–80 µM) are required to achieve 45–50% inhibition of XO [41]. While allopurinol is effective at relieving symptoms of gout, it may be a poor choice for targeting endothelial-associated XO-derived ROS generation.

Febuxostat is reported to have a *K*_*i*_ some 6000 times lower than allopurinol and does not react directly with the Mo-co site of XOR, but rather binds the pocket leading to the active site via electrostatic interaction thus blocking purine access. As such, it does not induce enzyme turn-over and unwanted production of O_2_^•-^ and H_2_O_2_ [40], [46], [47]. This makes febuxostat an ideal pharmaceutic for pathologic conditions where O_2_^•-^ and H_2_O_2_ generated by XO contribute to endothelial injury. The IC_50_ of febuxostat (4.4 nM) did increase 2.5-fold when XO was bound by GAGs, but this is significantly lower than the 22-fold increase observed with allopurinol (64 μM) [40]. Febuxostat completely inhibited O_2_^-•^ formation at or below 50 nM, a concentration well below the clinical *C*_*max*_ (15 μM) [40].

Whereas both pyrazolopyrimidine-based inhibitors and febuxostat are effective in reducing uric acid levels and oxidant generation by inhibiting XO, they are not without shortcomings. For example, clinical administration of allopurinol: 1) is limited to significantly reduced dosing in patients with preexisting renal disease, 2) is known to mediate substantive hypersensitivity issues which also limits dosage and 3) effects alternative purine catabolic pathways [15], [48]. While febuxostat has demonstrated superior specificity and potency to allo/oxypurinol, it too has been associated with negative clinical outcomes. For example, febuxostat administration has been associated with rhabdomyolysis in patients with chronic kidney disease (CKD) [49] and has been shown to be non-inferior to allopurinol regarding rates of adverse cardiovascular events where all-cause mortality and cardiovascular mortality were greater in febuxostat-treated patients than patients treated with allopurinol [50]. When taken together, the issues related to currently available FDA-approved XO inhibition approaches may indeed limit the potential for their effective use in hemolytic disease, affirming the need for alternative approaches to alter XO activity.

## 6 XOR, ROS and hemolytic diseases

**Fig. 2:**
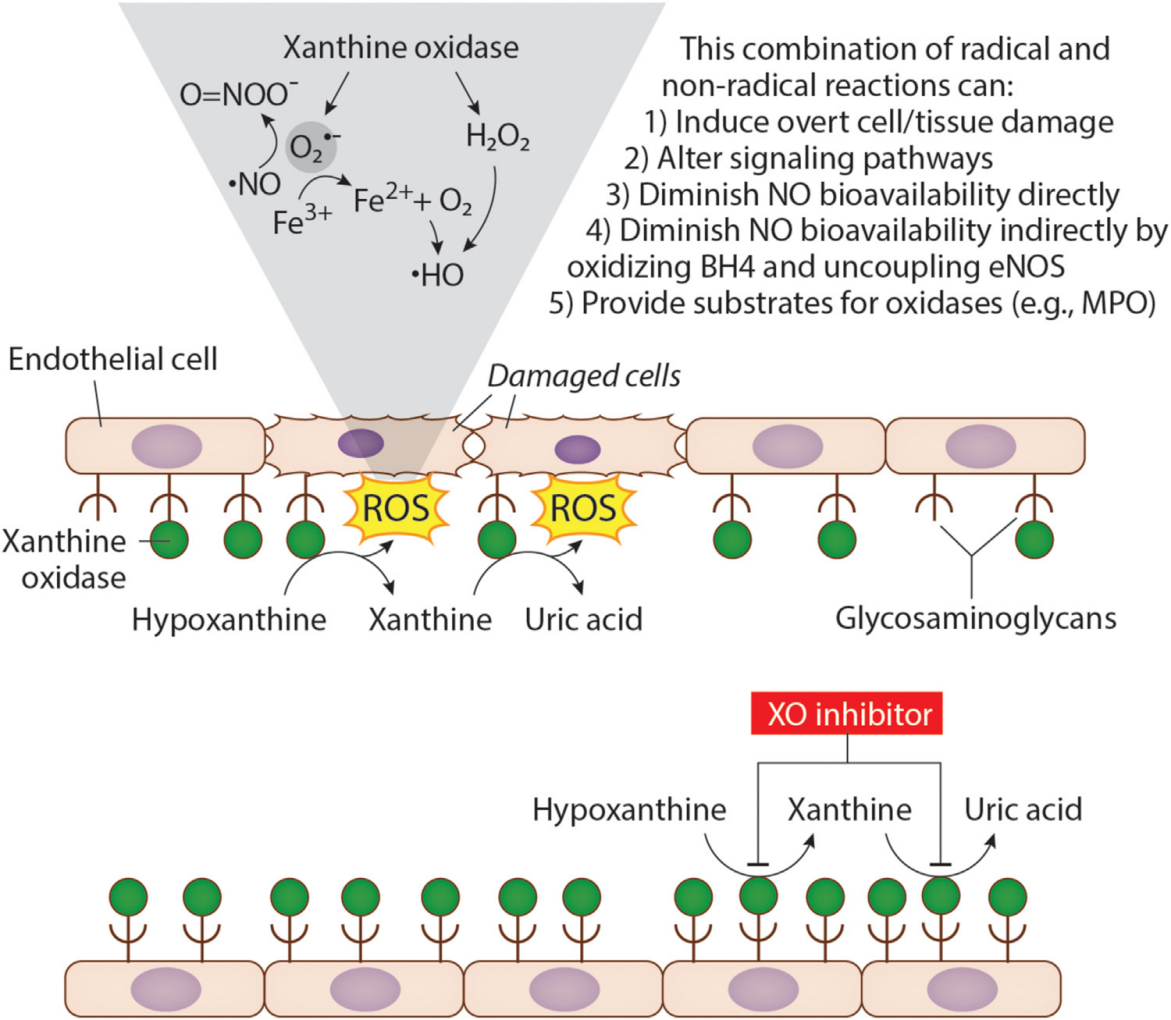
Xanthine oxidase (XO) released from the liver can bind to the surface of endothelial cells via interactions with glycosaminoglycans (GAGs). As hypoxanthine is converted to xanthine, and xanthine to uric acid by XO, reactive oxygen species (ROS) are generated at the surface of endothelial cells. XO produces either O_2_^•-^ or H_2_O_2_ which can lead to production of ONOO^-^ or ^•^HO (top). Treatment with an XO inhibitor such as febuxostat inhibits XO and blocks the conversion of hypoxanthine to xanthine and xanthine to uric acid preventing ROS production and damage at the endothelial cell surface (bottom).

Upregulation of XDH has been associated with hypoxia-induced vaso-occlusive crisis in SCD mice and humans [12], [51]. Aslan and colleagues demonstrated that ischemia/reperfusion injury in SCD mice resulted in release of XDH from the liver into the circulation, rapid conversion to XO via plasma proteases, immobilization on endothelial GAGs, and represented a major source of O_2_^•-^ and H_2_O_2_ in the vascular compartment [12], as depicted in Fig. 2. In this setting, XO-derived oxidants can alter cell signaling processes as well as induce overt damage [12]. For example, diminution in ^•^NO-mediated signaling (e.g. vasodilatory response) can result from the diffusion-limited reaction between XO-derived O_2_^•-^ and ^•^NO to form O=NOO^-^. In addition, both XO-derived O_2_^•-^ and H_2_O_2_ can mediate protein and lipid oxidation which can affect vascular homeostasis by also altering signaling pathways directly (e.g. oxidation of eNOS-associated BH_4_) or indirectly via disruption of cellular membrane integrity. Evidence for the contribution of XO to oxidant load is also revealed by substantively elevated circulating XO in SCD patients compared to healthy controls [51].

Hepatic ischemic injury and hypoxia, in mice and rats, also increase XDH release from the liver into the vasculature and induce rapid conversion of XDH to XO during reperfusion [52], [53]. Due to the relatively high circulating half-life of XO, significant damage can be done to the endothelium due to XO-dependent oxidant generation [52]. Elevated XO levels were observed in diseases involving liver injury such as hepatitis, jaundice, and chronic renal failure (secondary effects on the liver) [52]. Of particular interest is that elevated levels of circulating XO are reported to positively associate with chronic liver disease (cirrhosis, chronic hepatitis and cholestatic disorders); yet, they do not correlate with indices of liver damage, suggesting a signaling event alternative to hepatocellular damage is operative, and thus may drive upregulation of XDH with subsequent release into the circulation [54]. However, patients with virus-related cirrhosis demonstrated significantly enhanced levels of circulating XO that did positively correlate with the extent of liver damage as indicated by assessment of ALT leaving the field a bit unclear at present [55].

More specifically related to “hemolytic” disease are patients with malaria and/or sepsis. Asymptomatic malaria patients demonstrate a two-fold elevation in circulating XO levels compared to controls whereas patients hospitalized with severe malaria have plasma XO levels over 3.25-fold greater than asymptomatic individuals and thus 6.5-fold greater than controls [4]. Similarly, upon diagnosis, sepsis patients who did not subsequently survive presented with significantly elevated plasma (2.2-fold) XO activity [5]. However, 24 h post diagnosis these non-survivors displayed less plasma XO activity than survivors; thus, indicating a temporal relationship regarding circulating XO activity and the severity of the disease as well as the overall outcome. Regardless of survival outcomes, plasma XO activity correlated positively with indices of oxidant stress including protein carbonylation and lipid peroxidation. This principle was also seen in a rodent model of experimental sepsis where treatment with allopurinol and/or metal chelation therapy with desferrioxamine significantly abrogated the abundance of biomolecular free radicals indicating critical contributions from both XO and Fenton-type reactions stemming from RBC lysis [56].

While it is clear that there are contributory roles for XO in hemolytic disease, it is equally clear that the mechanisms underpinning specific signaling events leading to upregulation of XDH, triggers to release XDH into the circulation and the subsequent impact of circulating XO remain to be defined. For example, it has been suggested that TLR-4 signaling serves to elevate XOR levels [57]; yet, there are no reports revealing empirical evidence that this is the case. On the other hand, it has been reported that XO-derived ROS were essential for TLR4-induced NFAT5 (nuclear factor of activated T cells) activation of murine macrophages; again, leaving the field a bit unclear [58]. This gap in our current understanding of the linkage between heme overload and upregulation of XDH affirms the need for further study.

Hx is a natural antioxidant that binds heme as it is released during hemolysis and targets the complex to the liver parenchymal cells for degradation and recycling of the Hx and iron [59]. However, increased nitration of Hx during inflammation can diminish the protective antioxidant properties during heme toxicity [59]. Erythrocytes undergo a significant amount of oxidative stress and ROS production and thus require a number of enzymatic and non-enzymatic antioxidants including superoxide dismutase (SOD), catalase (CAT), glutathione peroxidase (GPx), glutathione reductase (GR), glutathione (GSH) and vitamins E and C [60]. SCD hemoglobin generates twice as much O_2_^-•^, H_2_O_2_ and HO^•^ compared to normal hemoglobin, and thus have increased antioxidant function often resulting in antioxidant deficiencies [60]. Antioxidant therapeutic approaches are being examined as an option for treating SCD because they can be administered at low cost through food and can be administered in combination with hydroxyurea to decrease oxidative stress [61].

## 7 Summary and conclusions

The high levels of hemolysis associated with hemolytic diseases, cause a saturation of protective pathways resulting in an increase in O_2_^•-^, H_2_O_2_ and ^•^HO generation and activation of inflammatory pathways [2], [8]. XO is one mechanism of ROS generation that has been observed in elevated circulating levels in murine models of hemolytic disease and clinically. Circulating XO can significantly contribute to the oxidant load in the vascular compartment by binding to the endothelial glycocalyx [34], [35]. In this setting XO is resistant to inhibition by clinically applicable concentrations of allo/oxypurinol and thus may serve to amplify the oxidant load derived from heme-related reactions and thus exacerbate an existing oxidant assault. However, the signaling events that link elevation in heme concentration and upregulation/export of cellular XDH are not defined. Further exploration of these signaling events will provide a clearer understanding of the role of XOR in hemolytic disease and likely improve clinical practices and outcomes.
